# Neutrophil/lymphocyte ratio predicts chemotherapy outcomes in patients with advanced colorectal cancer

**DOI:** 10.1038/bjc.2011.100

**Published:** 2011-03-29

**Authors:** W Chua, K A Charles, V E Baracos, S J Clarke

**Affiliations:** 1 Sydney Cancer Centre, Concord Repatriation General Hospital, Hospital Road, Concord, New South Wales 2139, Australia; 2 Faculty of Medicine, University of Sydney, Sydney, New South Wales, Australia; 3 School of Medical Sciences (Pharmacology) and Bosch Institute, University of Sydney, Sydney, New South Wales, Australia; 4 Department of Oncology, University of Alberta, Edmonton, Alberta, Canada

**Keywords:** colorectal cancer, prognosis, neutrophil/lymphocyte ratio, cancer-associated inflammation

## Abstract

**Background:**

Advances in the treatment of metastatic colorectal cancer (mCRC) in the last decade have significantly improved survival; however, simple biomarkers to predict response or toxicity have not been identified, which are applicable to all community oncology settings worldwide. The use of inflammatory markers based on differential white-cell counts, such as the neutrophil/lymphocyte ratio (NLR), may be simple and readily available biomarkers.

**Methods:**

Clinical information and baseline laboratory parameters were available for 349 patients, from two independent cohorts, with unresectable mCRC receiving first-line palliative chemotherapy. Associations between baseline prognostic variables, including inflammatory markers such as the NLR and tumour response, progression and survival were investigated.

**Results:**

In the training cohort, combination-agent chemotherapy (*P*=0.001) and NLR⩽5 (*P*=0.003) were associated with improved clinical benefit. The ECOG performance status ⩾1 (*P*=0.002), NLR>5 (*P*=0.01), hypoalbuminaemia (*P*=0.03) and single-agent chemotherapy (*P*<0.0001) were associated with increased risk of progression. The ECOG performance status ⩾1 (*P*=0.004) and NLR>5 (*P*=0.002) predicted worse overall survival (OS). The NLR was confirmed to independently predict OS in the validation cohort (*P*<0.0001). Normalisation of the NLR after one cycle of chemotherapy in a subset of patients resulted in improved progression-free survival (*P*=0.012).

**Conclusion:**

These results have highlighted NLR as a potentially useful clinical biomarker of systemic inflammatory response in predicting clinically meaningful outcomes in two independent cohorts. Results of this study have also confirmed the importance of a chronic systemic inflammatory response influencing clinical outcomes in patients with mCRC.

Colorectal cancer (CRC) is the third leading cause of worldwide cancer mortality after lung and stomach cancer and is responsible for 639 000 deaths or 1.1% of total deaths (World Health Organisation, 2004). There have been major advances in the treatment of metastatic CRC (mCRC) in the last 10–15 years, involving the introduction of new cytotoxic and molecular targeted therapies. However, use of these newer treatments result in increased toxicities and are prohibitively expensive. Hence, there is a need for accurate predictors of outcomes from treatment, in particular, in identifying those patients who are more likely to benefit by being assisted in rationalising increasingly expensive treatments, especially in under-resourced communities.

Tumour development and growth occurs as a result of interactions among the tumour, host-derived stromal tissues including blood vessels and host immune/inflammatory cells (see Figure 1), with chronic inflammation having an important role in cancer development and progression (Balkwill and Mantovani, 2010; Coussens and Werb, 2002). Lymphocytic infiltration in primary colorectal tumour tissue with different lymphocyte subpopulations has been investigated as potential prognostic factors (Pagès *et al*, 2005; Galon *et al*, 2006). This chronic inflammatory state also has effects on normal tissues, including the liver, resulting in an ongoing release of ‘acute-phase proteins’ that may be used to monitor this process. Current prognostication in advanced CRC, as in other malignancies, involves a poorly defined combination of clinical experience with the use of relatively crude and subjective covariates, such as performance status, with few markers in clinical practice apart from the use of *k-ras* mutation status and treatment with epidermal growth-factor receptor inhibitors (Bokemeyer *et al*, 2009; Koopman *et al*, 2009; Van Cutsem *et al*, 2009; Chua *et al*, 2010).

Over the last 10 years, laboratory markers of a systemic inflammatory response, including plasma C-reactive protein concentration (CRP), hypoalbuminaemia and Glasgow Prognostic Score (GPS, which combines CRP and albumin), and absolute white cell or its components (neutrophils, neutrophil/lymphocyte ratios (NLRs) and platelet/lymphocyte ratios (PLRs)) have been investigated as prognostic and predictive markers in different cancer populations, with the best evidence for their use demonstrated in surgical patients with CRC (Roxburgh and McMillan, 2010). Emerging evidence suggests that elevated baseline levels of CRP (Hilmy *et al*, 2005; Crumley *et al*, 2006a; Mitsuru *et al*, 2009; Johnson *et al*, 2010), abnormal GPS scores (Forrest *et al*, 2003; Crumley *et al*, 2006b; McMillan, 2009) and elevated NLR (Yamanaka *et al*, 2007; Halazun *et al*, 2008, 2009; Liu *et al*, 2010) or PLR (Smith *et al*, 2009) are not only negatively correlated with outcome after surgical resection but also in those with inoperable cancers. These inflammation scores based on readily available and inexpensive tests could potentially be ideal biomarkers of outcome in patients with mCRC.

Evidence for the use of these inflammatory markers as direct predictors of outcome in patients with advanced malignancy receiving first-line chemotherapy is lacking. Two recent studies have highlighted the use of the systemic inflammatory response in predicting survival (Teramukai *et al*, 2009) and toxicity (Arieta *et al*, 2010) in patients receiving chemotherapy for advanced non-small-cell lung cancer. Neutrophilia has been shown to be an adverse prognostic factor in patients receiving first-line oxaliplatin-based chemotherapy for CRC (Michael *et al*, 2006). An elevated NLR in CRC patients with liver-only colorectal metastases receiving neoadjuvant chemotherapy before surgical resection of liver metastases predicted worse survival (Kishi *et al*, 2009). In addition, those patients in whom NLR normalised after chemotherapy had significantly improved 1-, 3- and 5-year survival similar to patients with NLR⩽5 at baseline. These data suggest that NLR may be a readily available and useful biomarker for monitoring early response and prognosis with chemotherapy for CRC (Kishi *et al*, 2009). The aims of the current study were to investigate (1) NLR in predicting treatment response, toxicity and survival in medical patients receiving first-line chemotherapy for advanced CRC in a training set; (2) validating the results from the training cohort in a separate community patient cohort; and (3) assessing the impact of normalisation of NLR for monitoring early response during chemotherapy.

## Patients and methods

### Study population

In total, there were 349 patients with available clinical information and baseline laboratory parameters. The training set consisted of 171 patients enrolled in first-line chemotherapy trials at the Sydney Cancer Centre for advanced CRC between 1999 and 2007. The independent validation set included 178 patients from a community-based clinical database in the province of Alberta and included patients referred to medial oncology units at the Cross Cancer Institute who received first-line chemotherapy for advanced CRC between 2004 and 2007 (Prado *et al*, 2008). Table 1 lists the comparative baseline clinical information and laboratory parameters for both cohorts before chemotherapy commencement.

### Methods

Baseline clinical information and biochemical evaluation, including full blood count (neutrophils, lymphocytes, haemoglobin and platelets) and albumin before chemotherapy commencement, were collected in a database for patients in both the training and validation sets. Alkaline phosphatase was also collected in the training set. Prognostic variables with >10% missing data were not included in the analysis. Differential white-cell counts (neutrophils and lymphocytes) were also collected for patients before cycle 2 of chemotherapy. Response rates, dates of progression and survival were available for patients in the training set; however, only survival data were available for patients in the validation cohort. Dates of death were followed up by the investigators through hospital records, local Cancer Registries or phone contact through patient relatives, local medical practitioners and palliative-care services. Patients were consented to undergo analyses before commencing chemotherapy, and the study was approved by institutional research ethics committees in both Sydney and Edmonton.

### Statistical analysis

Statistical analyses were performed using SPSS Graduate Version 17.0 (IBM Corporation 2010, Somers, NY, USA). Response rates were determined according to criteria determined by individual clinical trials, RECIST criteria. Clinical response was defined as either complete or partial response and non-response as either stable or progressive disease. Clinical benefit was defined as complete response, partial response and stable disease and no benefit as progressive disease alone. Progression-free survival (PFS) was defined as the date of commencing protocol treatment to the date of first progression or death from any cause without progression. Overall survival (OS) was defined as the date from the date of commencing protocol treatment to the date of death from any cause. The *χ*^2^-tests were used to test associations between variables of interest (grouped using standard thresholds) and clinical response or benefit. Multivariate modelling was used for calculation of hazard ratios and clinical response and benefit. The follow-up period commenced at the start of chemotherapy with the censor date of January 2010. Survival analysis was performed using the Kaplan–Meier method with log-rank test in univariate analyses. Cox regression analysis was used for multivariate survival analysis and for calculation of hazard ratios.

## Results

### Patient characteristics

Baseline clinical demographics and laboratory values for both training and validation sets are presented in Table 1. There were no differences in age and gender between the two cohorts. However, a significantly higher proportion of patients in the validation cohort had rectal cancer as the primary tumour site and had ECOG PS⩾1. The majority of patients in both cohorts received combination chemotherapy±a biological agent.

### Prognostic variables in training set

Table 2 shows the univariate analyses between prognostic variables of interest and clinical benefit, PFS and OS in the training set. At the time of analysis, all patients had progressed on chemotherapy and 169 patients were deceased. The overall clinical response (complete response and partial response) was 55% (93 out of 168 evaluable patients) and clinical benefit (complete response, partial response and stable disease) was 75% (128 out of 168 evaluable patients). The median PFS was 6.7 months (95% CI 5.6–7.8 months) and OS was 15.3 months (95% CI 12.4–18.2).

#### Clinical benefit and response

Younger age (⩽65 years old), ECOG performance status 0, absence of hypoalbuminaemia, normal alkaline phosphatase, low or normal neutrophil counts and NLR⩽5 were associated with improved clinical benefit (Table 2). Similarly, younger age (⩽65 years old), ECOG performance status 0 and NLR⩽5 were associated with improved clinical response. In addition, combination-agent chemotherapy was also associated with improved clinical response.

#### PFS and OS

**Figure 2:**
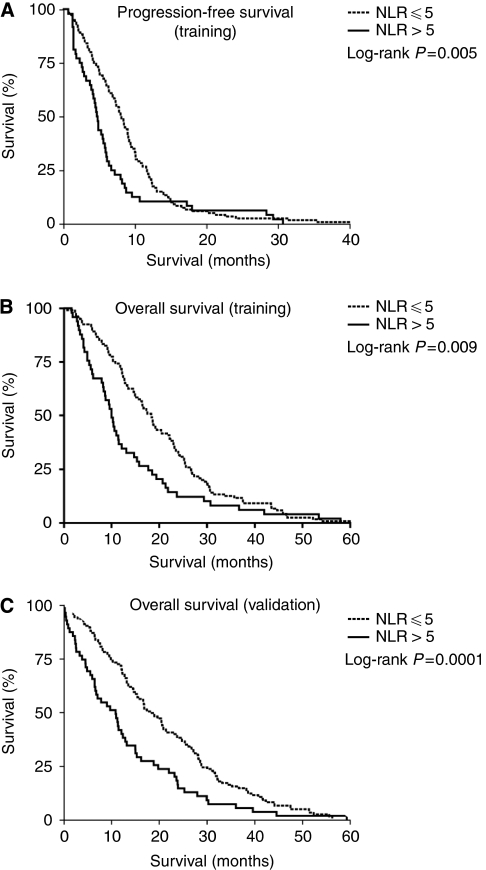
PFS according to NLR in (**A**) training cohort. OS according to NLR in **B** (training) and (**C**) validation cohorts of patients with mCRC treated with chemotherapy.

Variables predicting improved PFS included younger age, ECOG performance status 0, combination-agent chemotherapy, single site of metastasis, absence of neutrophilia or hypoalbuminaemia and NLR⩽5 (Table 2 and Figure 2A). The following variables were associated with improved OS: younger age, ECOG PS 0, combination-agent chemotherapy, absence of neutrophilia or anaemia. Hypoalbuminaemia, elevated alkaline phosphatase and NLR>5 were also significantly associated with worse OS (Table 2 and Figure 2B).

#### Multivariate analysis

In multivariate analysis performed in the training set (Table 3), combination-agent chemotherapy and NLR⩽5 were associated with improved clinical benefit. The ECOG performance status ⩾1, NLR>5, hypoalbuminaemia and single-agent chemotherapy were associated with increased risk of progression. The ECOG performance status ⩾1 and NLR>5 predicted worse OS.

#### Prognostic variables according to NLR

Table 4 summarises analysis of baseline characteristics and prognostic variables according to NLR groups. Patients with NLR>5 were more likely to suffer from hypoalbuminaemia (*P*-level <0.0001) and elevated alkaline phosphatase (*P*-level 0.008). The association between NLR and gender (*P*-level 0.06) and number of metastatic sites (*P*-level 0.05) approached statistical significance.

### NLR in validation cohort

At the time of analysis, 82% (146 out of 178) of patients were deceased. The median OS in this cohort was 16.8 months (95% CI 13.1–20.4 months). Independent predictors of survival from the training cohort (ECOG performance status and NLR) were tested in the validation cohort. The NLR was statistically significantly associated with OS (*P*-level <0.0001). Patients with NLR⩽5 had median OS of 19.1 months (95% CI 15.3–22.8) compared with patients with NLR>5 (median OS 11.3 months; 95%CI 8.3–14.3; Figure 2C). The ECOG performance status was not predictive of survival in this cohort (median OS for ECOG 0 was 21.5 months (95% CI 4.1–38.9) and PS⩾1 15.7 months (95% CI 13.1–18.3; *P*-level 0.15)).

### Normalisation of NLR pre-cycle 2 and correlation with PFS and OS (training cohort)

**Figure 3:**
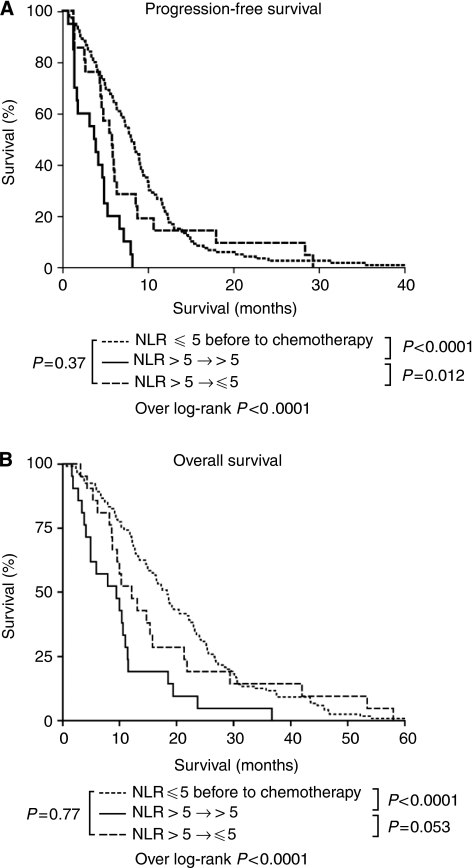
Changes in PFS (**A**) and OS (**B**) with normalisation of NLR in training cohort of mCRC treated with chemotherapy.

Patients were categorised into the following categories: (1) patients with NLR⩽5 at baseline (*n*=120; cohort 1), (2) NLR>5 at baseline and before cycle 2 of chemotherapy (*n*=21; cohort 2) and (3) NLR>5 at baseline with normalisation of NLR⩽5 before cycle 2 of chemotherapy (*n*=21; cohort 3). Patients with normalisation of NLR before cycle 2 of chemotherapy (cohort 3) had an improved PFS of 5.8 months (95% CI 4.1–7.5) compared with patients without normalisation of NLR pre-cycle 2 (cohort 2; median PFS 3.7 months; 95% CI 0.6–6.8 months; *P*-level 0.012; Figure 3A). Normalisation of NLR improved median OS from 9.4 months (cohort 2; 95% CI 3.2–15.5) to 12.1 months (cohort 3; 95% CI 7.3–16.8) in patients with a persistently elevated NLR, although this did not reach statistical significance (*P*-level 0.053; Figure 3B). Patients with normalised NLR before cycle 2 of chemotherapy (cohort 3) did not have significantly different median PFS (5.8 months (95% CI 4.1–7.5) *vs* 8.0 months (95% CI 6.9–9.0); *P*-level 0.37) or OS (12.1 months (95% CI 7.3–16.8) *vs* 18.3 months (95% CI 16.2–20.4); *P*-level 0.77) in comparison with patients with NLR⩽5 before chemotherapy commencement (cohort 1; Figures 3A and B). Normalisation of NLR before cycle 2 of chemotherapy was not performed in the validation cohort, as there was >10% of missing data for this patient group.

## Discussion

This is the first study, to our knowledge, to describe the use of NLR in a non-selected unresectable mCRC setting for patients receiving first-line palliative chemotherapy to provide useful information regarding prognostication, and the data have been validated in an independent community-based cohort. These results support the use of NLR as a marker of systemic inflammatory response and as an independent predictor of clinical benefit, progression and survival in patients receiving chemotherapy for mCRC. An NLR cutoff >5 was able to identify a subset of patients least likely to respond to chemotherapy (40 *vs* 16%) and those at higher risk of progression and death (HR 1.6 and 1.7, respectively). A cutoff score of 5 was chosen on the basis of previous studies (Halazun *et al*, 2008, 2009; Kishi *et al*, 2009) and this represents a simple measurement to use in clinical practice, although other cutoffs have been used (Duffy *et al*, 2006; Cho *et al*, 2009). This identifies ∼30% of CRC patients with a raised NLR receiving first-line chemotherapy in both cohorts and associated with shorter survival of up to 8 months. These results are highly clinically relevant in this increasingly common malignancy.

In addition, evidence for significantly improved outcomes with normalisation of NLR after the first cycle is promising for possible manipulation of the systemic inflammatory response through targeted anti-inflammatory mediators such as IL-6 blocking antibodies. If the use of NLR and normalistion of NLR after cycle 1 are confirmed, this would provide additional prognostic information for clinicians at an earlier time point before conventional staging with computed tomography scans and potentially identify a proportion of patients in whom further treatment may be futile. For example, in the training cohort, there was NLR normalisation after one cycle of chemotherapy in 50% (21 out of 42) of evaluable patients, which resulted in a 2-month PFS improvement (5.8 *vs* 3.7 months) compared with patients without NLR normalisation. These data will permit not only retrospective evaluations of established large cohorts with known outcome data to corroborate these findings but also to undertake correlation with molecular characteristics, such as microsatellite instability and *B-raf* mutations, which are associated with worse cancer outcomes.

The strengths in our training cohort were that patient data were retrospectively analysed from robust prospectively collected data through entry into clinical trials. As the patients were eligible for enrolment in a clinical trial, it is highly unlikely that the elevated NLR was due to other active inflammatory diseases or infection or were requiring high doses of steroids; however, these issues should be specifically assessed in future studies. Other independent predictive variables identified from the training cohort, such as performance status, use of combination chemotherapy and hypoalbuminaemia, have also been reported from previous studies and strengthens the case for this cohort being representative of a palliative mCRC population. The median OS in both cohorts (15.3 and 16.8 months in training and validation cohorts, respectively) are shorter than those reported using modern combination chemotherapy regimens, which have median OS upwards of 24 months. However, a significant proportion of the patients in both cohorts received single-agent chemotherapy, with patients enrolled in chemotherapy trials from as early as 1999. There were also significant baseline differences in the types of chemotherapy regimens between the Australian and Canadian cohorts. In the Canadian cohort, up to 29% of patients did not have the type of chemotherapy specified, which may account for some of the survival difference between the two cohorts. The validation cohort in this study failed to identify performance status as an independent prognosticator, which, although surprising, may reflect the community-based origins of this group. However, in both cohorts, the proportion of patients with NLR>5 was surprisingly consistent between the two cohorts (29 and 31%). In spite of differences between the cohorts, NLR remained an independent prognosticator and may reflect that it is an even more robust and accurate prognosticator than performance status alone. The heterogeneity of treatment regimens used could be criticised; however, this is probably more reflective of day-to-day clinical practice.

The NLR is a simple, readily available and robust laboratory variable. Other authors have advocated the use of GPS or a modified GPS, based on albumin and CRP levels, and validated its use as a prognostic variable particularly in the pre-operative setting. Two studies have reported the use of GPS in patients receiving chemotherapy for mCRC and gastro-oesophageal malignancies (Crumley *et al*, 2008; Ishizuka *et al*, 2009). However, this assessment is complicated by the requirement for an additional blood test to measure CRP levels, which may not be readily available as was in the case of both our training and validation sets. The NLR, as a continuous variable, may also be a more accurate and dynamic variable reflecting acute changes in the inflammatory state of a patient rather than GPS, which is applied as a static, categorical variable. The NLR and GPS have not been compared in the same population in CRC patients, and this comparison should be undertaken to discern whether these two indices are overlapping or additive as indicators of cancer-associated inflammation. In CRC, the use of NLR has previously been confirmed as an independent prognostic factor in a cohort of patients with liver-only colorectal metastases, the majority of whom proceeded to hepatic resection post chemotherapy (Kishi *et al*, 2009). Although this is an important subset of patients with mCRC, these patients would have been highly selected for surgical intervention and not representative of the majority of patients with mCRC. The findings in our study are not only consistent with this earlier report but also supports the use of NLR in a more generalised patient population receiving first-line chemotherapy both in a clinical trial and community setting. Although elevated NLR was correlated with the presence of hypoalbuminaemia and elevated alkaline phosphatase in this study (Table 4), other prognostic variables, such as performance status, site or extent of disease, were relatively well-balanced between the high- and low-NLR groups, suggesting that NLR provides additional information than these other variables. The association of both raised NLR and hypoalbuminaemia is likely because of its role as a marker of systemic inflammation. The reasons for the correlation between alkaline phosphatase and NLR are unclear. Alkaline phosphatase may be a more accurate marker of the extent of liver involvement or indirectly related to systemic inflammation. The NLR has also been previously shown to independently predict outcomes in non-malignant disease, such as post-ST-segment elevation myocardial infarction (Núñez *et al*, 2008) and percutaneous coronary intervention (Duffy *et al*, 2006) in which the systemic inflammation response has been implicated as a major contributing factor. This adds credibility for the use of NLR as a potential biomarker of the systemic inflammatory response.

In recent years, there have been significant developments and discoveries in cancer genomics. The development of gene-expression-based arrays or examining germline single-nucleotide polymorphisms for defining prognosis or predicting response to therapy has limited clinical application even in the two most common malignancies, lung and breast cancers (Hartman *et al*, 2010; Subramanian and Simon, 2010). For example, Wacholder *et al* (2010) discovered that the inclusion of 10 common breast cancer genetic variants only modestly improved the performance of existing risk-assessment models in >11 000 patients, with little change in the predicted breast-cancer risk among most women, using currently available genetic information. These tests are also expensive and confined to use in developed countries, with limited application in under-resourced communities. A useful biomarker needs to be not only accurate and reproducible but also easily accessible. The prognostic importance of the systemic reaction to tumours has been relatively ignored in the quest for tumour-based molecular assessments of outcome. These data will encourage a re-evaluation of that approach.

These results have highlighted the use of a potential clinical biomarker of systemic inflammatory response in predicting clinically meaningful outcomes in two independent cohorts. In addition, results of the study have also confirmed the importance of a chronic systemic inflammatory response influencing clinical outcomes in patients with mCRC. Validation of these results in larger patient populations will allow many potential applications in the treatment of mCRC, a major cause of morbidity worldwide. Clinical applications include (1) prognostication and in-patient stratification in clinical trials, (2) as a marker of response to chemotherapy treatment and, more excitingly, (3) in identifying patients for possible interventions with anti-inflammatory mediators. The results of this study, we believe, strongly support the use of NLR in these settings, and more importantly, as a dynamic marker of interactions among tumour, host and the systemic inflammatory response.

## Figures and Tables

**Figure 1:**
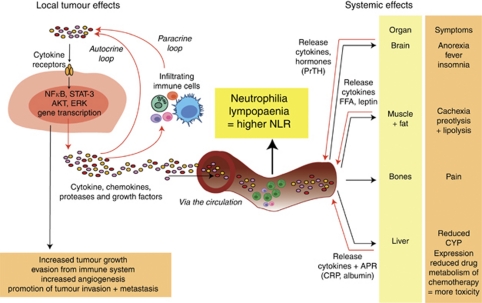
The effect of cytokines in the local tumour environment and systemic organs, and the clinical manifestations of these interactions. Red arrows indicate cytokines being released from either tumour or other organ.

**Table 1:** Baseline patient characteristics in the training and validation sets before commencement of chemotherapy

	**Training set (*n*=171)** **No. (%)**	**Validation set (*n*=178)** **No. (%)**	***P*-value**
Age, median (range)	61 (33–84)	63 (32–85)	0.40
Gender (M/F)	110/61 (64/36)	100/73 (56/44)	0.22
			
*Primary cancer site*	—a		
Colon	103 (64)	116 (65)	
Rectosigmoid	52 (32)	23 (13)	
Rectum	4 (3)	39 (22)	
Synchronous	2 (1)	0	<0.0001
			
*ECOG* *performance status*			
0	85 (50)	30 (17)	
1	81 (47)	96 (54)	
⩾2	5 (3)	52 (29)	<0.0001
			
*Chemotherapy regimen*			
Single agent only	43 (25)	34 (19)	
Combination chemotherapy±biologicals	128 (75)	92 (52)	
Unknown	0	52 (29)	<0.0001
			
*Number of sites*			
1	91 (53)	NA	__
>1	80 (47)		
			
*Baseline levels of prognostic factors*			
Albumin, median (range), g l^−1^	40 (27–48)	39 (20–47)	
Carcinoembryonic antigen, median (range)	38.6 (0.7–945)	NA	
Haemoglobin, median (range)	127 (82–162)	122 (78–170)	
Neutrophils, median (range)	5.5 (1.5–14.8)	5.1 (1.9–21.4)	
Lymphocytes, median (range)	1.4 (0.4–4.9)	1.5 (0.2–3.3)	
Neutrophil–lymphocyte ratio, median (range)	3.7 (1.0–30.8)	3.5 (0.9–74.5)	
⩽5	120 (71)	123 (69)	
>5	49 (29)	55 (31)	

Abbreviations: ECOG=Eastern Cooperative Oncology Group; F=female; M=male; NA=not available.

a Missing data for 10 patients (training set).

**Table 2:** Univariate analysis of clinicopathological factors, inflammatory response and response rate, PFS and OS in training set

		**Clinical benefit**		**PFS**		**OS**	
**Variable**	**Total no. (%)**	**Clinical benefit (%)^a^**	***P*-value**	**Survival (months)** **Median (95% CI)**	***P*-value**	**Survival (months)** **Median (95 %CI)**	***P*-value**
*Age, years*							
⩽65	113 (66)	90 (81)		7.4 (5.8–9.0)		17.5 (14.5–20.8)	0.03
>65	58 (34)	38 (67)	0.04	5.5 (4.3–6.7)	0.05	12.1 (10.9–13.3)	
							
*Gender*							
Male	110 (64)	81 (74)		6.1 (4.7–7.6)		15.8 (12.6–19.0)	0.85
Female	61 (36)	47 (80)	0.44	7.3 (5.1–9.5)	0.13	14.6 (10.0–19.2)	
							
*ECOG PS*							
0	85 (50)	69 (84)		9.0 (8.2–9.7)		18.5 (16.1–21.0)	0.003
⩾1	86 (50)	59 (69)	0.02	4.9 (4.0–5.7)	0.001	11.5 (9.3–13.7)	
							
*Primary site*							
Colon	103 (64)	76 (75)		6.3 (5.1–7.5)		12.9 (10.8–15.1)	
Rectosigmoid junction	52 (32)	40 (77)		8.5 (6.9–10.1)		18.3 (16.9–19.8)	
Rectum	4 (3)	3 (75)		5.0 (0–12.6)		4.3 (9.0–25.6)	
Synchronous	2 (1)	1 (50)	0.86	3.9 (—)	0.21	11.4 (—)	0.51
							
*Chemotherapy*							
Single	43 (25)	23 (54)		3.9 (2.3–5.5)		11.0 (8.2–13.9)	
Combination	128 (75)	105 (84)	0.07	8.0 (6.9–9.1)	<0.0001	17.5 (14.5–20.6)	0.01
							
*Number of sites*							
1	91 (53)	72 (82)		8.4 (7.2–9.6)		17.5 (14.3–20.6)	
>1	80 (47)	56 (70)	0.07	5.0 (4.0–6.0)	0.06	12.3 (7.4–17.3)	0.18
							
*Neutrophil count*							
<ULN	133 (78)	105 (80)		7.8 (6.7–9.0)		17.4 (15.5–19.4)	
⩾ULN	38 (22)	23 (62)	0.02	4.8 (3.4–6.2)	0.02	9.4 (6.6–12.1)	0.004
							
*Haemoglobin*							
⩾LLN	79 (46)	61 (77)		7.7 (5.9–9.4)		17.5 (15.5–19.6)	
<LLN	92 (54)	67 (78)	0.77	6.0 (4.8–7.2)	0.58	12.7 (10.1–15.4)	0.03
							
*Albumin*							
>LLN	117 (69)	32 (63)		4.9 (3.9–5.9)		18.3 (16.5–20.1)	
⩽LLN	53 (31)	96 (83)	0.005	8.0 (6.9–9.0)	0.002	10.4 (7.7–13.2)	0.002
							
*Alkaline phosphatase*							
<ULN	96 (56)	77 (82)		8.0 (6.9–9.1)		18.3 (15.6–21.1)	
⩾ULN	74 (44)	50 (69)	0.04	5.5 (4.3–6.7)	0.25	11.5 (9.5–13.5)	0.007
							
*NLR*							
⩽5	120 (71)	99 (84)		8.0 (6.9–9.0)		18.3 (16.2–20.4)	
>5	49 (29)	29 (60)	0.001	4.7 (4.1–5.3)	0.005	10.0 (8.6–11.5)	0.009

Abbreviations: CI=confidence interval; ECOG=Eastern Cooperative Oncology Group; OS=overall survival; LLN=lower limits of normal; NLR=neutrophil/lymphocyte ratio; PFS=progression-free survival; PS=performance status; ULN=upper limits of normal.

a Missing data (<10%) for some prognostic variables.

**Table 3:** Multivariate analysis of prognostic factors of clinical response, PFS and OS (training set)

	**Prognostic variable**	**Hazard ratio (95% CI)**	***P*-level**
Clinical benefit	*Chemotherapy type*		
	Single agent	1	
	Combination	4.0 (1.8–9.2)	0.001
			
	*NLR*		
	>5	1	
	⩽5	3.4 (1.5–7.8)	0.003
			
PFS	*ECOG performance status*		
	0	1	
	1 or more	1.5 (1.1–2.1)	0.02
			
	*NLR*		
	⩽5	1	
	>5	1.6 (1.1–2.2)	0.01
			
	*Albumin*		
	⩾LLN	1	
	<LLN	1.5 (1.0–2.1)	0.03
			
	*Chemotherapy type*		
	Combination	1	
	Single agent	2.8 (1.9–4.0)	<0.0001
			
OS	*ECOG performance status*		
	0	1	
	1 or more	1.6 (1.2–2.2)	0.004
			
	*NLR*		
	⩽5	1	
	>5	1.7 (1.2–2.5)	0.002

Abbreviations: CI=confidence interval; ECOG=Eastern Cooperative Oncology Group; OS=overall survival; LLN=lower limits of normal; NLR=neutrophil/lymphocyte ratio; PFS=progression-free survival.

**Table 4:** Baseline characteristics according to NLR

**Variable**	**NLR⩽5**	**NLR>5**	***P*-level**
Age (median)	61	61	0.32
			
*Gender*			
Male	72 (60%)	37 (76%)	
Female	48 (40%)	12 (24%)	0.06
			
*ECOG performance status*			
0	69 (58%)	24 (49%)	
1 and 2	60 (50%)	25 (51%)	0.90
			
*Number of metastatic sites*			
1	69 (58%)	20 (41%)	
>1	51 (42%)	29 (59%)	0.05
			
*Primary site*			
Colon	70 (62%)	31 (67%)	
Rectum and rectosigmoid junction	42 (37%)	14 (30%)	
Synchronous	1 (1%)	1 (1%)	0.61
			
*Site of metastases*			
Liver	96 (80%)	44 (90%)	0.13
Lung	37 (31%)	17 (35%)	0.63
Other	43 (36%)	21 (43%)	0.39
			
*Anaemia*			
Present	64 (53%)	28 (57%)	
Absent	56 (47%)	21 (43%)	0.65
			
*Hypoalbuminaemia*			
Present	26 (22%)	26 (53%)	
Absent	93 (78%)	23 (47%)	<0.0001
			
*Alkaline phosphatase levels*			
<ULN	75 (63%)	20 (41%)	
⩾ULN	44 (37%)	29 (59%)	0.008

Abbreviations: ECOG=Eastern Cooperative Oncology Group; NLR=neutrophil/lymphocyte ratio; ULN=upper limits of normal.
